# How Well Do Self-Supervised Models Transfer to Medical Imaging?

**DOI:** 10.3390/jimaging8120320

**Published:** 2022-12-01

**Authors:** Jonah Anton, Liam Castelli, Mun Fai Chan, Mathilde Outters, Wan Hee Tang, Venus Cheung, Pancham Shukla, Rahee Walambe, Ketan Kotecha

**Affiliations:** 1 Department of Computing, Imperial College London, London SW7 2AZ, UK; 2 Symbiosis Institute of Technology, Symbiosis International University, Pune 412115, India; 3 Symbiosis Centre for Applied Artificial Intelligence, Symbiosis International University, Pune 412115, India

**Keywords:** self-supervised learning, medical imaging, image classification, BYOL, MoCo, PIRL, SWaV, SimCLR

## Abstract

Self-supervised learning approaches have seen success transferring between similar medical imaging datasets, however there has been no large scale attempt to compare the transferability of self-supervised models against each other on medical images. In this study, we compare the generalisability of seven self-supervised models, two of which were trained in-domain, against supervised baselines across eight different medical datasets. We find that ImageNet pretrained self-supervised models are more generalisable than their supervised counterparts, scoring up to 10% better on medical classification tasks. The two in-domain pretrained models outperformed other models by over 20% on in-domain tasks, however they suffered significant loss of accuracy on all other tasks. Our investigation of the feature representations suggests that this trend may be due to the models learning to focus too heavily on specific areas.

## 1. Introduction

In recent years, machine learning algorithms like convolutional neural networks (CNNs) have been massively successful across a variety of computer vision tasks, such as classification [1,2,3], object localization and detection [4,5,6,7] and segmentation [8]. These algorithms have found success in medical imaging, with models achieving results comparable to trained medical professionals in a variety of areas, such as chest radiography, fundus imaging and dermatology [9,10,11].

Most of these models are trained within the supervised learning paradigm, where the model is given a dataset of image-label pairs. To achieve strong performance when training CNNs—which often have millions of learnable parameters—with supervised learning, the training dataset must be extremely large [12]. The curation of massive, human-annotated datasets, however, is prohibitively time-consuming and expensive. This annotation process is particularly challenging within the medical domain, as often expert personnel is required for the interpretation of the medical images and there are privacy concerns when using patients’ medical data [10,13,14].

Self-supervised learning (SSL), in contrast to supervised learning, does away with the necessity for annotated data, using the data itself as the supervisory signal for learning rich feature representations, allowing it to leverage large, unlabelled datasets during training. The learned features are then optimised for a task downstream, finetuning the features with supervised-learning on a smaller, target task-specific dataset [15]. The algorithm by which the features are learned is known as the pretext task. State-of-the-art pretext tasks build the representation space on the core idea of similarity, where representations for images which encode similar semantic content are embedded near one another in feature space, and far apart for images which encode different semantic content. The state-of-the-art pretext tasks do however differ in their exact methodology, and a brief overview is provided in Section 2 of this paper.

Self-supervised learning, therefore, is particularly promising for medical image analysis, where unlabelled datasets are abundant and labelled data is scarce. Previous works using SSL for medical imaging, surveyed in [14], tend to directly pretrain and then finetune on the same dataset [16,17]. However, we instead seek to investigate the transferability of learned image features from pretrained networks on ImageNet [18] to medical datasets. The motivation behind this is fourfold:

i. ImageNet supervised pretrained features have been found to transfer very poorly to medical imaging tasks [19]. However, in [20], it is demonstrated that ImageNet self-supervised pretrained features tend to transfer much better than their supervised counterparts to a variety of downstream tasks, some with large domain shifts from ImageNet. It remains to be seen, however, if this improved transfer performance also applies to medical imaging.
ii. Medical images differ significantly in their structure to the natural images found in ImageNet, which are non-cluttered and have a clear global object. Many medical images are extremely unstructured, such as skin lesions [21]. Even those with a clearer object-like structure, for example X-rays, have characteristic signatures associated with the different categorical labels (which in a medical context often correspond to different pathologies) that tend to be minute local textural variations [19]. Medical image analysis has therefore proven to be a difficult task for deep learning models. Consequently it provides a strong test of the generalisability and robustness of the features learned by self-supervised pretraining.
iii. Refs. [16,17] find improved performance over supervised ImageNet pretrained features through performing self-supervised pretraining, as well as finetuning, on the target medical dataset. However, it is unclear whether such domain-specific self-supervised pretrained models significantly outperform similarly pretrained ImageNet alternatives.
iv. Taking publicly available pretrained models and finetuning them is significantly less computationally expensive than pretraining from scratch, and therefore allows us to perform interesting, and, to the best of our knowledge, new analysis without massive resources.

In this work, a detailed analysis of a broad spectrum of experiments is carried out. The transfer performance of pre-trained models is analysed to understand the generalisability of both supervised and self-supervised (domain-specific as well as ImageNet pretrained) models to a variety of medical imaging datasets.

All code is open-sourced (https://github.com/jonahanton/SSL_medicalimaging (accessed on 30 September 2022)). The datasets and models considered can be found in Section 4.1 and Section 4.2, respectively.

In total, 3 ImageNet supervised pretrained models, 5 ImageNet self-supervised pretrained models, 2 domain-specific self-supervised pretrained models, and 9 medical datasets, covering chest X-rays, breast cancer histology images, and retinal fundus images are considered. We seek specifically to answer the following research questions:

Q1. How do supervised, self-supervised methods compare for medical downstream tasks? A: We find that self-supervised methods are able to outperform supervised methods across the vast majority of medical downstream tasks with few-shot and many-shot linear learning. Of the self-supervised methods, the Bootstrap Your Own Latent (BYOL) method was found to be the best overall performer. More careful treatment of hyperparameters is needed to come to conclusive results about many-shot finetune learning.
Q2. Is there a clear benefit to domain-specific self-supervised pretraining?
  A: Yes, provided the downstream task is also in the same domain. Domain-specific pretrained self-supervised models, trained on chest X-rays, were much better than self-supervised or supervised models on two of the four chest X-ray datasets. However, it is observed that the performance drops off significantly as the domain of the dataset shifts, and hence even the slight shift in domain of the remaining two chest X-ray datasets was enough to drastically reduce the classification accuracy.
Q3. What information is encoded in the pretrained features?
  A: The domain-specific models appear to encode only very specific areas for in-domain data with a significantly lower attentive diffusion than the other types of models. Due to the nature of disease manifestations occurring as small texture differences in medical images, this hyperfocus on key areas may help explain its improved performance compared to the more holistic approach of SSL and supervised models.

### Contribution and Novelty

This paper makes the following key contributions:

1. To the best of our knowledge, this is the first large scale comparison of pretrained SSL models to standard pretrained models for transferring to medical images.
2. This is also one of the first attempts to directly compare the transferability of SSL models pretrained on ImageNet vs. those pretrained on a medical domain specific task for a variety of different medical imaging datasets, allowing us to directly quantify the benefits of both approaches.
3. Finally, we are able to show through the analysis of the encoded features how in domain pretraining leads to a more focused feature extraction than standard ImageNet pretraining, which can massively boost performance for in domain tasks at the expense of generalisability.

The remainder of this paper is organised as follows. In Section 2, a brief overview of self-supervised learning and the current state-of-the-art is given. In Section 3, the related works are discussed, and in Section 4 we present the methodology by which the analysis is performed. Our results and discussion are presented in Section 5, and we conclude in Section 6 with a summary and comment on possible future developments.

## 2. Overview of Self-Supervised Learning

Self-supervised learning is a paradigm in which the unlabelled data itself is used as a supervisory signal for machine learning. As this type of learning does not require labels, it is particularly valuable for the medical domain where creating annotated medical image datasets can be prohibitively expensive [14]. While self-supervised learning approaches can be broadly split into three categories (generative, contrastive and predictive), the state-of-the-art SSL methods are dominated by contrastive approaches [14]. Contrastive learning develops effective representations through analysing the similarity of input pairs.

Contrastive learning is a type of instance discrimination, which treats augmented versions of the same image as a similar pair, contrasted against the similarity of all other possible inputs. By introducing a variety of strong data augmentations, invariances can be learned which allow the models to be robust to changes in orientation, colour, scale and other transformations. However, these approaches are generally limited by the need to have significant numbers of dissimilar pairs for the model to be effective [20].

There are multiple solutions to the above problem. The Simple Contrastive Learning (SimCLR) framework [22] uses large batch sizes, allowing direct comparison of instances across the batch, while Pretext-invariant Representation Learning (PIRL) [23] instead stores dissimilar instances in a memory bank. PIRL also heavily leverages a Jigsaw pretext task, where the model is trained to learn invariance to random shuffling of image patches within the inputs. The Momentum Contrast (MoCo) [24] model uses a momentum encoder and a queue to produce contrastive instances, with the added benefit of smoother updates and smaller memory requirements.

An alternative approach is introduced in the Bring Your Own Latent (BYOL) approach [25], which allows the model to learn without explicitly sampling negative pairs. Instead, two networks, an online network and a target network, where the target network is a moving average of the online network, are used in parallel on the same input after different augmentations [25]. The online network predicts the feature projection of the target network.

Swapping Assignment between Views (SWAV) [26] is one of the most popular SSL learning approaches that does not rely on instance discrimination, and while the initial steps of the learning framework are similar to those in SimCLR, the feature vectors undergo (soft) clustering online to a set of learnable prototypes. These soft cluster assignments are known as *codes*. Similar to BYOL, it is required that the feature vector of one view can compute the code for the other view of the same image, and vice versa, allowing the model to learn feature representations that are invariant to the chosen data augmentations [26].

The above is a brief summary of SSL, focussing specifically on the approaches used in this paper. See [27] for a more comprehensive discussion and survey of SSL techniques.

## 3. Related Work

### 3.1. Transfer Performance of Self-Supervised Models

Ericsson et al. [20] evaluate how well (ImageNet) self-supervised pretrained models transfer to a variety of downstream tasks (including few-shot recognition, object detection and dense prediction), specifically in comparison to one another and (ImageNet) supervised pretrained models. They find that self-supervised pretrained models outperform supervised ones across almost all tasks considered, indicating the generalisability of self-supervised features. Our work builds on this, specifically looking at transfer performance applied to classification tasks on medical datasets, further investigating the robustness of self-supervised models. Other works [28,29] have investigated the transfer performance of self-supervised pretrained models to medical datasets, but they have either only used a small number of models [28] or a single domain [29]. Our work attempts to further the analysis started in these papers, specifically looking at how a large number of SSL models compare across a variety of medical domains.

### 3.2. Domain-Specific Self-Supervised Learning for Medical Image Analysis

Many works that apply SSL to medical images transfer the model within domain to a related downstream task [14,16,17,30]. Sowrirajan et al. [16] perform self-supervised pretraining with MoCo on the CheXpert chest X-ray dataset [31], and then finetune on CheXpert with different fractions of labelled training data. They find that their model outperforms a supervised ImageNet pretrained model, both on CheXpert and Shenzhen-CXR, a small chest X-ray dataset [32]. Sriram et al. [17] also uses MoCo on CheXpert, however first performs supervised pretraining on the chest X-ray dataset MIMIC-CXR-JPG [33] before applying the model for COVID-19 prognosis. They found that the SSL pretrained model transferred better to the COVID-19 dataset than supervised alternatives [17]. In this work, we also do in-domain transfers with the above two models, however they are applied to four chest X-ray datasets and their performances are compared against one another to further investigate their transferability within domain. Navarro et al. [34] investigate the generalisability of self-supervised and supervised models-trained on multi-organ segmentation - to unseen kidney segmentation data. However, the multi-organ segmentation dataset also included kidney segmentations. In our work, we further investigate model performance on datasets that are of a significant domain shift from the training data.

### 3.3. Generalisability of Self-Supervised Features

Ref. [35] investigates the generalisability of self-supervised features, specifically looking at features learned through contrastive instance discrimination. They suggest that supervised features are less generalisable than self-supervised ones due to forced minimisation of intra-class variation. Supervised learning methods force the model to assign the same categorical label to all instances within the same class, potentially ignoring unique information related to each instance. This can lead to poor transfer performance if there exists any misalignment between pretraining and the downstream task. We continue this analysis by investigating whether this robustness of self-supervised features also translates to a more holistic and accurate modelling of medical images.

## 4. Materials and Methods

### 4.1. Models

The following pretrained models are considered.

**Supervised:** ResNet-50 [2], ResNet-18 [2], and DenseNet-121 [3]. All these models were pretrained on the ImageNet training set (1.3M images) in a supervised learning fashion, and are available from the PyTorch Torchvision library [36].

**Self-Supervised:** SimCLR-v1 [22], MoCo-v2 [24,37], PIRL [23], SwAV [26], and BYOL [25]. All of the above models use ResNet-50 backbone feature extractors and have been pretrained on the ImageNet training set. In the following text, unless explicitly stated otherwise, we use the term self-supervised models only to refer to SSL methods that were pretrained on ImageNet.

**Domain-specific Self-Supervised:** We consider two domain-specific self-supervised pretrained models, MIMIC-CheXpert [17] and MoCo-CXR [16], both of which were pretrained on chest X-ray datasets. The authors provide three pretrained MIMIC-CheXpert models, all of which use a DenseNet-121 backbone, differing in the learning rate used during MoCo pretraining, $\in 10^{\left\{-2,-1,0\right\}}$. All three models are considered, however often only the results for the best performing one is presented. MoCo-CXR is available with two different backbone architectures, DenseNet-121 and ResNet-18. Both are considered in this work.

All of these models have different training schedules, hyperparameters and data augmentations applied. Exact details can be found in the original publications. For all models the weights are made available by the original authors, except for with PIRL, for which the ones provided by the PyContrast GitHub repository (https://github.com/HobbitLong/PyContrast, accessed on 5 April 2022) are used. The majority of the models use a ResNet-50 backbone, except for the domain-specific self-supervised models (MIMIC-CheXpert, MoCo-CXR), which use the DenseNet-121 and ResNet-18 architectures. Therefore, we evaluate the corresponding supervised pretrained architectures for comparison. For all models the inputs are normalized with the mean and standard deviation of the ImageNet training set, except for SimCLR-v1, as the model has not been trained on normalised inputs, and MIMIC-CheXpert, which expects histogram normalisation [17].

### 4.2. Datasets

8 different medical datasets are considered, covering a range of medical domains.

#### 4.2.1. Preliminaries

We explore the comparative transfer performance of self-supervised models pretrained on the CheXpert dataset [31] to self-supervised and supervised models pretrained on ImageNet. As part of this comparison, the classification performance across a variety of different medical datasets is investigated. We classify the datasets into 2 broad classes: in-domain versus out-of-domain (relative to the domain-specific self-supervised models).

In-domain datasets comprise the chest X-ray datasets. Three additional chest X-ray datasets are explored on top of CheXpert, which we consider to be small, in-domain, distributional shifts: ChestX-ray14 [38], Montgomery-CXR [32], and Shenzhen-CXR [32]. ChestX-ray14 is the smallest shift, as it has a similar range of pathology labels to CheXpert (7 overlapping labels). Montgomery and Shenzhen are slightly larger shifts, as they only contain images of a single pathology, Tuberculosis, rather than multiple, and Tuberculosis is not present in CheXpert.

We have four out-of-domain datasets that include all non chest X-ray images: iChallenge-PM [39], iChallenge-AMD [39], EyePACS, and BACH [40]. The first three are all retinal fundus images, which, similar to chest X-rays, project a 3D object onto a 2D plane, and hence contain no depth information [10]. All three are regular RGB images, although taken with different types of cameras. The BACH dataset contains stained breast histology images and represent a similarly large shift from X-rays. The images from this dataset contain false colouring due to the staining agent, and are of microscopic tissue, a medical imaging domain which has not been represented in any of the other datasets [40].

**Figure 1:**
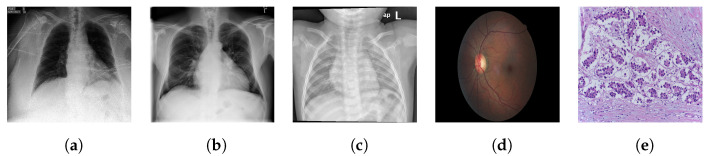
Example images from the (**a**) CheXpert, (**b**) ChestX-ray14, (**c**) Shenzhen-CXR, (**d**) EyePACS, (**e**) BACH datasets.

Dataset specifics, including the number of images per dataset, can be found in Table 1, and a few example images are shown in Figure 1. Terminology pertaining to our categorisation of datasets and models can be found in Table 2.

#### 4.2.2. Preprocessing

All images are converted to RGB. For greyscale images, like those in the chest X-ray images, the images are stacked to give a valid three-channel output as in [41].

The labels are converted to binary where possible. For the CheXpert dataset, which is multi-label, this is done through many-to-one, as in [30]. The most common pathology (Pleural Effusion, 40.34% of all images) is given a positive label, and all other pathologies and a lack of disease are labelled as negative. For datasets with textual labels, like Montgomery and Shenzhen, [32] is followed, treating any abnormal X-ray as a positive label. A similar approach is taken with the iChallenge-PM dataset, combining the normal and high myopia classes into a single negative label, while the pathological myopia class is treated as the positive label, as suggested in [39]. For BACH, a multi-class categorical dataset, the labels are treated as ordinal and converted to a single number between 0 and 4, as in [42]. ChestX-ray14, which is multi-label, is treated as in [43], with only the images with a single pathology being used for multi-class classification.

### 4.3. Evaluation Setup

Unless otherwise stated, the design decisions made in [20] are followed to allow for fair comparison of results.

#### 4.3.1. Few-Shot Learning

Many of the datasets considered contain only a small number of images: Shenzhen-CXR, Montgomery-CXR, ChestX-ray14, BACH, iChallenge-AMD and iChallenge-PM all include under 1000 images (Table 1). Finetuning a pretrained model on the training set of these datasets would lead to severe overfitting, resulting in very poor classification performance on the test set. Therefore, for these datasets we are in the (cross-domain) “few-shot” regime, where the model is tasked with learning to categorize a new set of classes, disjoint from those seen during training, with very few examples per class [43].

Following from [20], we use the technique of Prototypical Networks [44] for few-shot recognition. During each training episode, the model is presented with *N* randomly selected examples from *K* randomly selected classes. This constitutes the *support set* $S={\left\{x_{i},y_{i}\right\}}_{i=1}^{K\times N}$, and is known as "*K-way N-shot*" few-shot learning. Prototypes $c_{k}$ for each class *k* are then learned as the centroid of the embedded features from the model for that class,

(1) $$c_{k}=\frac{1}{N}\sum_{\left(x_{i},y_{i}\right)\in S_{k}}f_{\phi }\left(x_{i}\right),$$

where $f_{\phi }\left(\cdot \right)$ is the model. A *query set* is then randomly selected, constituting of $N_{Q}$ samples for each class *k*, and classification is performed for each query by finding the nearest class prototype.

For all pretrained models and each of the 8 datasets we consider 2-way 20-shot transfer, except for the datasets ChestX-ray14 and EyePACS, for which 5-way 20-shot transfer is used since these contain more than 2 classes. 600 episodes are randomly sampled and the average accuracy is reported along with 95% confidence intervals. The query set always contains 15 images per class ($N_{Q}=15$).

#### 4.3.2. Many-Shot Learning

We choose to perform many-shot recognition on the datasets CheXpert and EyePACS, which contain over 200,000 and 30,000 images, respectively, and so are both firmly in the “many-shot” regime. Given a pretrained model $f_{\phi }\left(\cdot \right)$, many-shot recognition is performed through two different methods: *linear* or *finetune*.

**Linear:** The pretrained model is frozen and leveraged as a fixed feature extractor. A multinomial logistic regression is fitted on top of the fixed features,

(2) $$P\left(y=c_{i}|x\right)=\frac{e^{w_{i}\cdot x}}{\sum_{k=1}^{K}e^{w_{k}\cdot x}},$$

where x is the feature representation, $\left\{w_{1},\ldots ,w_{K}\right\}$ are a learned set of weights, $w_{i}\in R^{d}$ where *d* is the dimensionality of the extracted features (*d* = 2048 for a ResNet-50 backbone, 1024 for DenseNet-121, 512 for ResNet-18), and $\left\{c_{1},\ldots ,c_{K}\right\}$ are the set of class labels. Following from [20], the $\ell_{2}$ regularization constant is selected on the validation set considering 45 logarithmically spaced values between $10^{-6}$ and $10^{5}$. The logistic regression model is then retrained on the entire training and validation set with the selected $\ell_{2}$ regularization constant, and evaluated on the test set. No data augmentation is applied during training, except for bicubic resampling to 224 pixels followed by a centre crop of 224 × 224.

**Finetune:** All pretrained parameters are refitted, along with an attached linear classification head, in a supervised learning fashion on the target dataset. The model is trained for a maximum of 5000 steps, optimised using Stochastic Gradient Descent (SGD) with Nesterov momentum, with the momentum parameter set to 0.9. Early stopping is implemented with a patience of 3 using the classification accuracy on the validation set as the relevant metric, checking every 200 steps. We train with a batch size of 64 for all models, except for those which use a DenseNet-121 backbone, which train with a batch size of 16 (due to GPU memory constraints). Due to resource constraints, the learning rate is fixed to $10^{-2}$ and the weight decay to $10^{-8}$. Random crop with resize and horizontal flip data augmentations are applied during training.

### 4.4. Analysis Tools

To investigate the representations learned, a variety of tools are developed. These tools provide further insight into what the model prioritizes in its representations.

#### 4.4.1. Saliency Maps

In order to investigate where in an image the models focus, activation saliency maps are computed. Following from [20], an occlusion-based saliency method is used, where a 10 × 10 mask is passed over the images (resized to 242 × 242). The root relative squared error (RRSE) is computed between the original image feature and the occluded image feature, which is added to the attention value for each pixel every time it is occluded by the mask. The attention values are averaged over all times a pixel is occluded ($10^{2}$), and the image is then cropped to 224 × 224 to ensure all pixels are occluded the same number of times. A high attention value for a given pixel means that the extracted feature vector is strongly perturbed by the occlusion of that pixel, meaning that the network is highly sensitive to this region. The attentive diffusion values are also computed, which correspond to the proportion of the saliency map with value above its mean [20]. A high attentive diffusion value corresponds to a broad focus. We use this occlusion-based saliency method instead of more popular methods to extract saliency maps, such as Grad-CAM [45], since it is independent of the choice of task [20].

#### 4.4.2. Deep Image Prior Reconstructions

We investigate what information from the original images is retained in the extracted features from the different models. To do so, we use the methodology as in [35] and observe the ability to reconstruct original images from the features. The feature inversion algorithm relies on the Deep Image Prior [46] to invert the pretrained networks’ feature maps. Given an original image, an encoder-decoder network is trained to map a fixed noise *z* to an image *x*, whose representation is as close as possible to the original image’s representation in the embedding space.

To quantify the quality of the reconstructed images, the Learned Perceptual Image Patch Similarity (LPIPS) perceptual distance metric from [47] is used, which measures the distance between two images’ deep embeddings across the layers of a pretrained deep neural network (specifically the AlexNet, SqueezeNet, and VGG architectures).

#### 4.4.3. Invariances

As part of our investigation, we would like to know why certain models transfer better than others. Hence, we consider the impact transformation invariances have on the generalisability of the model.

We measure the invariance of feature vectors, from different datasets, to different data transformations. Following from the method proposed in [48], the invariance of images *x* in a dataset *D*, to a set of transformations $t_{\theta }$ parameterised by parameters θ∈Θ, with associated features $f_{\phi }\left(x\right)$, is measured by the cosine similarity

(3) $$L_{f}^{T_{\Theta }}\left(D\right)=\frac{1}{\left|D||\Theta \right|}\sum_{x\in D,\theta \in \Theta }\frac{z\cdot z_{t_{\theta }}}{\left||z|||\right|z_{t_{\theta }}\left|\right|},$$

where $T_{\Theta }={\left\{t_{\theta }\right\}}_{\theta \in \Theta }$,

(4) $$z=L(\bar{f_{\phi }}-f_{\phi }\left(x\right)),$$

(5) $$z_{t_{\theta }}=L\left(\bar{f_{\phi }}-f_{\phi }\left(t_{\theta }\left(x\right)\right)\right),$$

*L* is the Cholesky decomposition of the feature covariance matrix $\Sigma^{-1}=LL^{T}$, $\bar{f_{\phi }}$ is the mean feature, and we average over all images x∈D and θ∈Θ. A cosine similarity close to 1 implies strong invariance, whereas one close to 0 implies little to no invariance. As an example, for horizontal flip invariance, the set Θ corresponds to *True* if a horizontal flip transformation is applied or *False* if not. A variety of synthetic data transformations are considered, including rotation, horizontal flip, and hue transforms, as well as *multi-view* invariance. Multi-view invariance is unique to the CheXpert and EyePACS datasets, since both contain more than one perspective of the same object being imaged. In this case, the transformation *t* is not parameterised by a set of parameters θ, but instead the scalar product in Equation (3) is considered to be between features extracted from an image pair, $x_{1}$ and $x_{2}$, corresponding to two different perspectives of the same object. In practice, to compute the mean feature vector $f_{\phi }$ and covariance matrix Σ, we consider 1000 randomly chosen images from the dataset *D* (or the entire dataset in the case that |D|<1000), and to compute the cosine similarity we average over 100 randomly sampled images.

## 5. Results

### 5.1. How Do Supervised, Self-Supervised Methods Compare for Medical Downstream Tasks?

**Figure 2:**
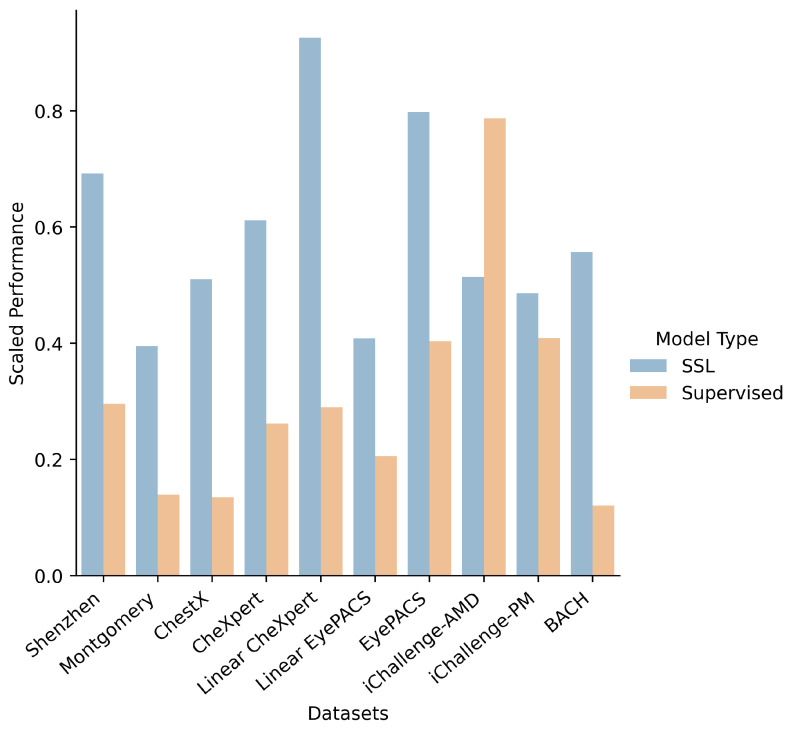
Bar chart of model transfer performance on different downstream tasks. SSL (blue) refers to the average over the self-supervised models (SimCLR, MoCo, SwAV, BYOL, PIRL) and Supervised (orange) refers to the average of the supervised models (ResNet-50, ResNet-18, DenseNet-121). For each downstream task, all results are scaled between 0 and 1 (across the SSL and Supervised models - not including the domain-specific models).

The accuracy of the transferred models in the few-shot and many-shot regimes are given in Table 3 and Table 4, respectively. We find that, in general, the SSL models (pretrained on ImageNet) outperform their supervised pretrained counterparts across the majority of downstream tasks, as shown in Figure 2.

There are two notable exceptions to the above trend: the performance on iChallenge-AMD, and the performance on finetune CheXpert and EyePACS.

For iChallenge-AMD, we hypothesize that the improved performance for supervised models may be due to the nature of age-related macular degeneration (AMD). A key indicator of AMD is an abnormal macula, which is located in the centre of the retina. AMD containing fundus images therefore hold the pertinent image information in the central portion of the image. ImageNet images similarly have this central focus, which may allow for supervised models to transfer more effectively than they have for other medical imaging tasks.

The performance with finetuning is less surprising. Catastrophic forgetting of previously learned information can occur in neural networks due to their shared weights [49]. Without careful treatment of the network and hyperparameter tuning, the beneficial representations learned during pretraining can be forgotten. Due to resource constraints, thorough hyperparameter tuning is not conducted, which may explain the difference in performance.

**Figure 3:**
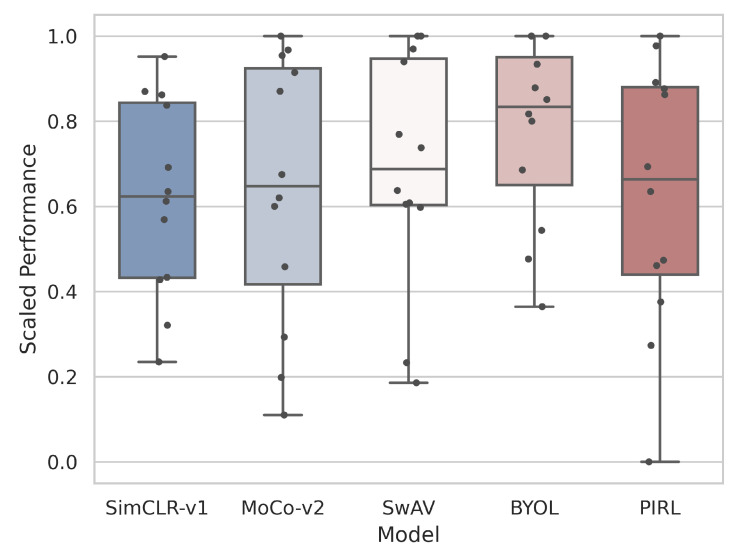
Box-and-whisker plot of model type (SSL models pretrained on ImageNet) against performance for few-shot recognition. Performance values are scaled between 0 and 1 for each dataset across all models. The performance on each dataset is plotted as a dot for each model.

To assess the impact of the specific SSL method applied, the various SSL models specifically pretrained on ImageNet are compared against each other in Figure 3. Overall, BYOL appears to be the most robust SSL method, achieving the highest average performance with small standard deviation.

Further, we consider if there exists any correlation with ImageNet accuracy and transfer performance. A linear fit is performed across all models for each dataset (Appendix D). We find there to be no significant correlations and suggest that this may be due to large differences between ImageNet and medical datasets. This is in line with [20], as they find that for large domain shifts, ImageNet performance is not indicative of transfer performance.

### 5.2. Is There a Clear Benefit to Domain-Specific Self-Supervised Pretraining?

**Figure 4:**
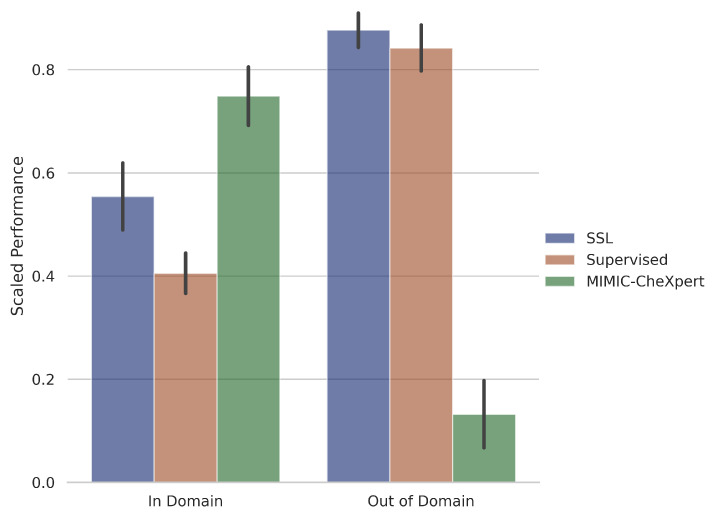
Performance (few-shot, linear, and finetune) on in-domain and out-of-domain datasets for the different model types, Self-supervised learning (pretrained on ImageNet), Supervised and MIMIC-CheXpert. In-domain datasets are comprised of CheXpert, ChestX, Montgomery and Shenzhen, while out-of-domain datasets are comprised of BACH, EyePACS, iChallenge-AMD, iChallenge-PM. Results are scaled between 0 and 1 (across all models), averaged over each model type, and then averaged over each dataset. Error bars correspond to 1σ variations across the individual models that are averaged over.

We classify models into 3 broad classes—supervised, self-supervised and domain-specific self-supervised. As discussed in Section 4.2.1, the medical datasets are classified as in-domain or out-of-domain, according to whether they are chest X-ray images. We plot the scaled performance of different classes of models on different types of datasets in Figure 4. We note that in this figure we only focus on MIMIC-CheXpert for the domain-specific self-supervised method, as we find significantly inferior performance with MoCo-CXR (Table 3 and Table 4). This trend is explored later in this section.

We observe that MIMIC-CheXpert outperforms supervised and self-supervised methods on in-domain datasets but suffers a significant deterioration in performance for out-of-domain datasets. This suggests a benefit that while there are benefits to domain-specific pretraining, they come at the expense of generalisability to out-of-domain datasets. This is likely due to the vast structural differences between medical image types and the monochromatic nature of chest X-ray images, which is causing the features learned from chest X-ray pretraining to generalise poorly. This result remains true but is less pronounced when we include the performance of MoCo-CXR in our in-domain self-supervised models.

**Figure 5:**
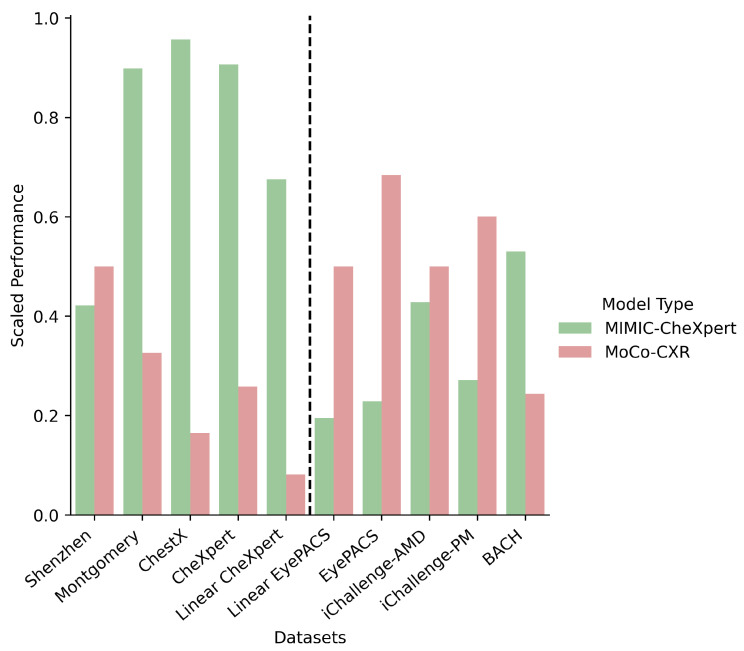
Bar chart of model transfer performance on different downstream tasks for the MIMIC-CheXpert (green) and MoCo-CXR (red) models. Results are scaled between 0 and 1. All in-domain datasets are located to the left of the dotted line.

Furthermore, the finding that specialised self-supervised training improves in-domain performance but worsens out-of-domain performance depends on how much in-domain pretraining is done. In Figure 5, the average performance of the MIMIC-CheXpert models is directly compared against the average performance of the MoCo-CXR models, and is found to perform significantly better in most in-domain datasets (Montgomery, ChestX, CheXpert) even though both models are trained on chest X-ray images. However, the MIMIC-CheXpert models are worse on out-of-domain datasets. This is likely due to the more extensive pretraining on chest X-rays for MIMIC-CheXpert. MoCo-CXR was trained only for 20 epochs using MoCo on CheXpert, compared to MIMIC-CheXpert, which was pretrained on MIMIC-JPG in a supervised manner for 10 epochs, followed by self-supervised training on MIMIC-CheXpert for 200 epochs.

From Table 3, we also note that performance of in-domain self-supervised models (MIMIC-CheXpert and MoCo-CXR) is actually worse than the other self-supervised and supervised models on the Shenzhen-CXR dataset, despite the fact that the Shenzhen-CXR dataset also contains chest X-ray images. The images from the Shenzhen-CXR dataset appear to have significant visual differences to those from the other chest X-ray datasets (compare, for example, Figure 1a–c). The domain shift from CheXpert to Shenzhen-CXR may, therefore, be too large for CheXpert pretraining to provide significant performance benefits over the ImageNet pretrained models.

In conclusion, we find that improvements in performance can be achieved through the use of domain-specific self-supervised pretraining. However, the features learned during this pretraining are not nearly as generalisable as those learned from ImageNet, and require the downstream dataset to not only be the same domain (i.e., chest X-rays) but also to be similar to the original dataset upon which the model was pretrained. A possible justification for this trend is explored in Section 5.3.

### 5.3. What Information Is Encoded in the Pretrained Features?

Deep image prior reconstructions are created for a single image from each dataset and for each model, and the associated perceptual distance metric is calculated for each of the three different architectures AlexNet, SqueezeNet, and VGG (see Table A4, Table A5, Table A6 in Appendix B). For each model, the average perceptual distance across the three reconstructions is plotted against the transfer accuracy for a given dataset, and a linear fit is performed (see Figure A3 in Appendix D).

**Figure 6:**
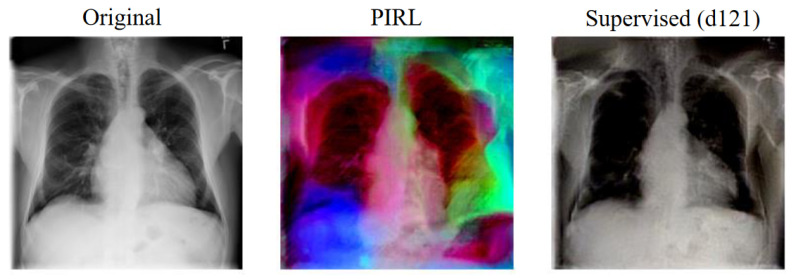
Deep image prior reconstructions for an image from the CheXpert dataset (**left**) for PIRL (**centre**) and Supervised DenseNet-121 (**right**). The original image is shown on the left for comparison.

A visual inspection of a few specific reconstructions in Figure 6 suggests that supervised pretrained models in general have better colour consistency to the original than self-supervised pretrained models, supporting the assertion made in [20]. However, we find that none of the linear regression fits are appropriate for the data, with no fit having a $\chi_{\nu }^{2}$ less than 1.5.

This at first glance appears counter-intuitive, since one would naively expect a strong correlation between reconstruction quality and transfer performance, since a better reconstruction implies a more robust feature representation. One possible explanation for the difference could be due to the nature of medical images, where different pathologies are often characterised by minor textural differences located in specific areas [19]. Hence, while reconstruction is important, it is the reconstruction of the key areas of the image which matter the most. Furthermore, perceptual distance, which attempts to quantify the similarity of images from a human perspective, may focus more on the general overall structures in the image than on the details, as humans do [47,50]. Hence, this may not be a particularly useful metric to assess how effective the feature representations of each model are for medical images, as the small variations in images are significantly more important than the general overall structure.

**Figure A2:**
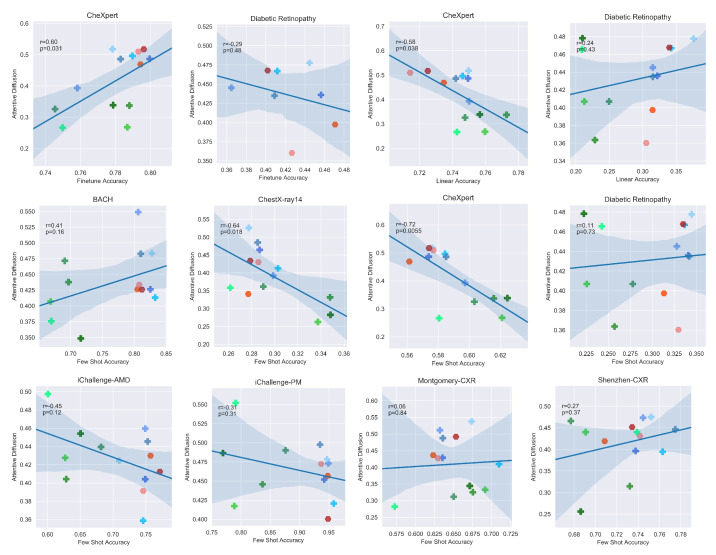
Attentive diffusion values for the reconstructed images plotted against transfer performance. Shown overlaid are the Pearson’s *r* correlation coefficients and associated *p* values.

**Figure A3:**
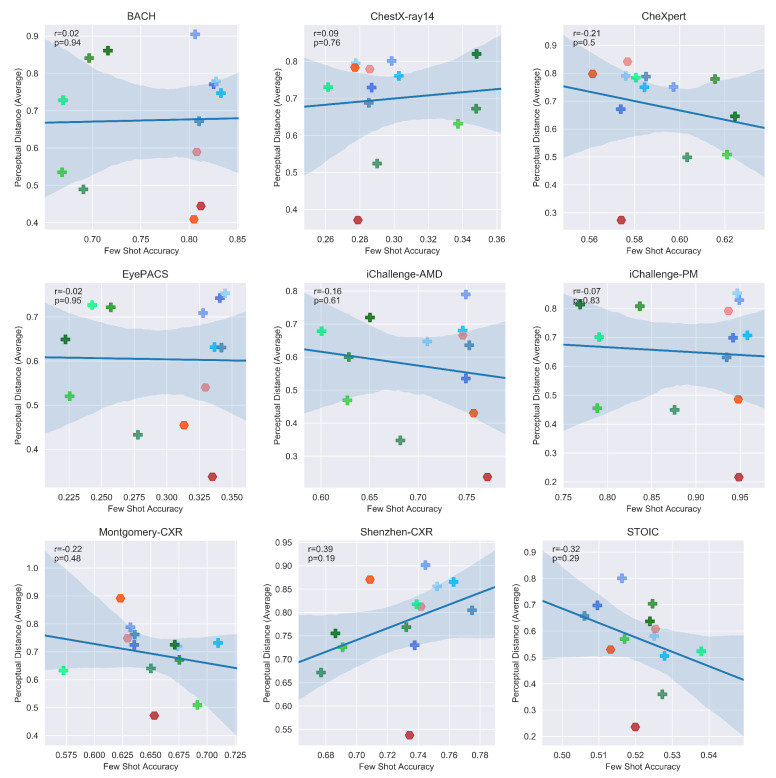
Perceptual distance values for the reconstructed images (averaged over the AlexNet, SqueezeNet, VGG architectures) plotted against transfer performance. Shown overlaid are the Pearson’s *r* correlation coefficients and associated *p* values.

**Figure A6:**
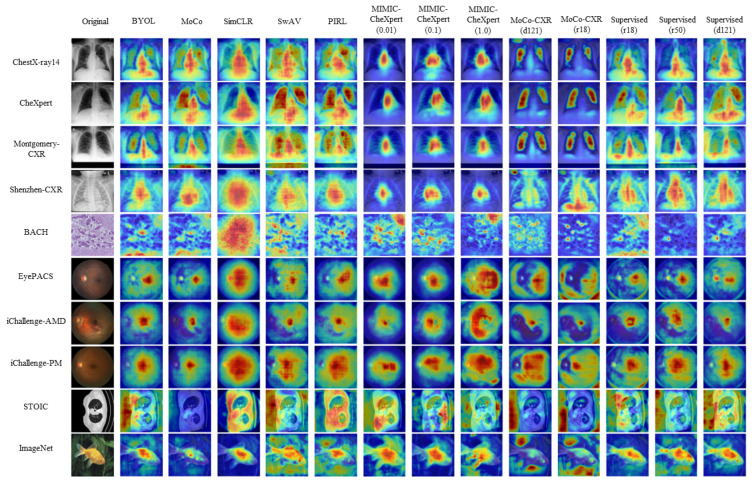
Saliency maps for all models on one image from each dataset.

Without image segmentation of diseased regions, it is difficult to directly assess the quality of the reconstructions in the key areas. However, the above discussion suggests that the best models will be highly focused on particular regions of the image, where the diseases tend to manifest. This can be explored through the saliency maps discussed in Section 4. Using the same images as the prior reconstructions, saliency maps are created for all possible dataset-model combinations (see Figure A6 in Appendix D) and the associated attentive diffusion is calculated (see Table A7 in Appendix C). The attentive diffusion of each model is plotted against transfer accuracy for a given dataset, and a linear fit is performed (see Figure A2 in Appendix D).

**Figure 7:**
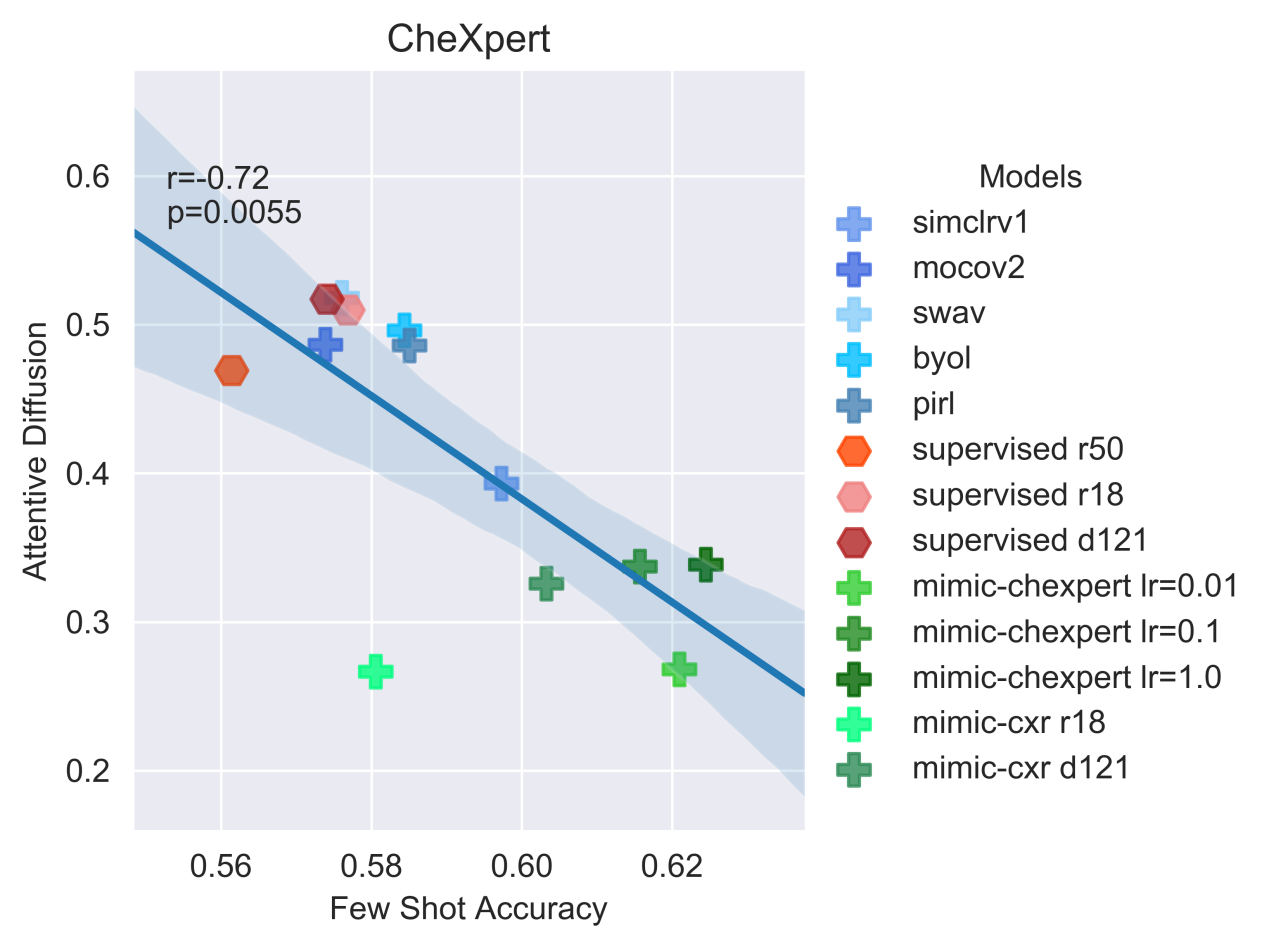
Few-shot accuracy on the CheXpert dataset plotted against attentive diffusion values for the reconstructed CheXpert image for all models. Shown overlaid are the Pearson’s *r* correlation coefficient and associated *p* value. A negative Pearson’s *r* close to −1 (with a low associated *p* value close to 0) implies a strong negative correlation.

When the images are in-domain (i.e., chest X-ray datasets) and classified by a model pretrained with domain-specific self-supervised methods, we find that there exists a clear linear relationship between attentive diffusion and transfer accuracy, as in Figure 7. This supports the hypothesis suggested earlier, that domain-specific models, which generally have the strongest performance on X-rays, focus exclusively on specific areas of the image while the supervised and SSL models focus more on the image holistically.

**Figure 8:**
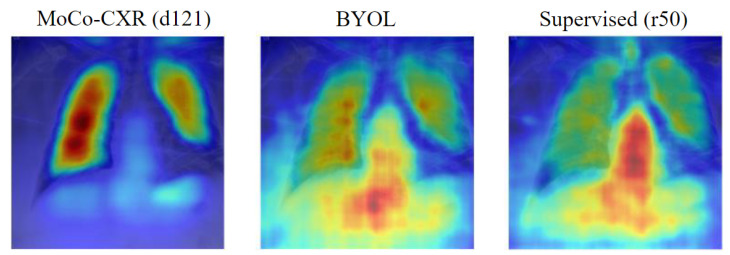
Saliency map for an image from the CheXpert dataset for the MoCo-CXR (**left**), BYOL (**centre**) and Supervised ResNet-50 (**right**) models. The three saliency maps have attentive diffusion values 0.33 (MoCo-CXR), 0.50 (BYOL), and 0.47 (Supervised r50).

This is further supported by the saliency plots, displayed in Figure 8, which show that the MoCo-CXR model focuses exclusively on the lung section of the X-ray, which is where most of the CheXpert pathologies manifest [38]. However, when the dataset is not a chest X-ray, there is no relation between attentive diffusion and transfer accuracy. We attribute this lack of a trend to a limitation of attentive diffusion as a metric; a low attentive diffusion implies that the model focuses on a particular area, however this does not check that this area is the important area of the image where the disease may manifest. Hence, while the domain-specific SSL models focus on the correct image regions, on outside-of-domain images they may focus on unimportant areas.

**Figure 9:**
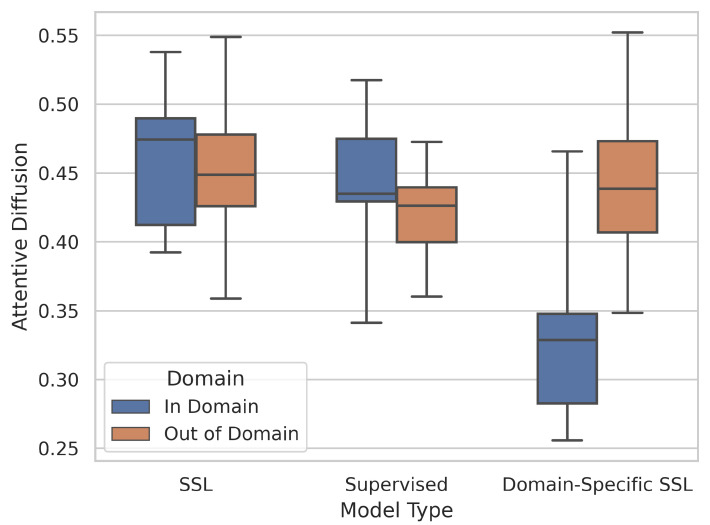
Box-and-whisker plot of model type (SSL, Supervised, and Domain-Specific SSL (MIMIC-CheXpert and MoCo-CXR)) against attentive diffusion values, split between in-domain (CheXpert, ChestX, Montgomery, Shenzhen) and out-of-domain (BACH, EyePACS, iChallenge-AMD, iChallenge-PM) datasets.

This observation further aids the analysis presented in Section 5.2. The benefits of in-domain training are significant, as the models learn to focus on the areas of the image which are most important to classifying the diseases. This claim is supported by Figure 9 which demonstrates that domain-specific models (MIMIC-CheXpert, MoCo-CXR) have a low attentive diffusion for in-domain datasets (the chest X-ray datasets) and thus focus on very specific image regions. However, this comes at the cost of generalisability. When applied to domain-shifted data, even if the domain shift is small (e.g., to Shenzhen-CXR), the model may over-focus on unimportant image regions. SSL models pretrained on ImageNet, in contrast, generally focus on a larger image region, which can allow them to adapt to significantly different domains as they view the image more holistically. This is demonstrated in the higher attentive diffusion for SSL methods (trained on ImageNet) which reveals that they tend to have a broader focus (Figure 9).

We also attempt to understand how well invariances have been encoded into the pretrained features. As shown in [48], during self-supervised pretraining, the model learns invariances to different data augmentations. We seek to answer whether these learned invariances transfer downstream to the target medical datasets, and if particular invariances are beneficial to specific medical downstream tasks. To quantify invariance to a given transformation, we use the cosine similarity metric as defined in Equation (3). We find that, in general, enforced invariances during pretraining do translate to invariances on target medical datasets. Consider, for example, MoCo-CXR, which achieves a horizontal flip invariance (cosine similarity) of 0.997 on CheXpert, the dataset it is pretrained on, and 0.778 on ChestX. Full results can be found in Appendix A. However, we generally find no significant correlation between invariance strength (cosine similarity) and transfer performance for any of the datasets with any of the transformations considered, suggesting a more nuanced relationship between learned invariances and feature generalisability.

One exception to that is for CheXpert, where we find a clear correlation between multi-view invariance and transfer performance (Pearson’s *r* of 0.82, which has an associated *p* value of 0.0068). This result is consistent with those found by Azizi et al. [30], who find that when they enforced this invariance to different views of the same underlying pathology during self-supervised pretraining, with a technique they call Multi-Instance Contrastive Learning (MICLe), there is a significant performance gain on CheXpert classification. We note that the multi-view invariance cosine similarity values themselves are all very low (below 0.025), however we suggest that this is likely due to this specific invariance not being enforced during pretraining.

## 6. Conclusions

Extending the work of [20], we have conducted the first systematic evaluation of SSL performance on medical datasets. This is not only novel but significant given the intrinsic difficulty of medical image analysis as well as the expensive annotation costs in medical data which reduces the scope for supervised training. Our work finds that (1) self-supervised models (trained on ImageNet) generally outperform supervised models (trained on ImageNet) in medical datasets due to the improved generalisability of self-supervised pretrained features. Unlike in [20], we find that (2) ImageNet performance does not correspond to downstream performance on medical datasets. In addition, there is no clear best-performing model (supervised/self-supervised) across all datasets, and although BYOL is best on average it is outperformed on multiple datasets by other models. Hence, one will still need a systematic evaluation of different methods on the specific medical dataset to determine the best method for that dataset.

Apart from considering “off-the-shelf” supervised and self-supervised methods trained on ImageNet, we also investigate the performance of in-domain self-supervised methods which have been trained on chest X-rays. We find that (3) in-domain self-supervised training offers a benefit only on in-domain datasets, but performance deteriorates significantly when out of that domain, highlighting a substantial decrease in generalisability. This is true even if there may be just a small shift in domain. Hence, we (4) recommend in-domain self-supervised training if one has access to a large section of unlabelled data, and only if one is certain that the downstream data is in a very similar domain as the pretraining data.

Finally, we also attempt to investigate the reasons behind the differing downstream performances by considering differences in feature representations. We find (5) no significant correlation between feature reconstruction and downstream performance, suggesting that pathologies are characterised more by minor changes rather than the overall structure of an image. Using saliency plots, (6) we find that domain-specific self-supervised methods focus on a small and salient area of the image, but focus incorrectly when outside of domain. This explains their improved in-domain performance at the expense of poor generalisability due to attentive overfitting [51]. Finally, we find that (7) enforced invariances during self-supervised pretraining translates to invariances on target medical datasets, but that has no correlation with transfer performance in general.

Our work has several limitations. First, we focus only on classification problems for our downstream task—ideally, we hope to extend our analysis to a variety of different downstream tasks. In particular, segmentation is a critical problem in medical imaging [14] and we are in the midst of including further analysis on this problem. Second, due to limited computational resources and time, we are unable to perform hyperparameter tuning for our finetuning tasks. Due to GPU memory issues, we also have to limit the batch size to 16 for DenseNet-121 architectures during finetuning (while batch sizes for all other architectures are 64). Therefore, we have been careful in drawing firm conclusions based on our finetune results.

Future works may consider investigating whether the trends noted in this report extend to other medical imaging fields not considered. Further, in this analysis we only consider in-domain pretraining on chest X-rays. It would therefore be interesting to see if in-domain pretraining on a different type of medical dataset, e.g., retinal fundus images, would lead to more generalisable features.

## Figures and Tables

**Table 1:** The type, number of images, and number of classes for each medical dataset considered. Type CXR corresponds to Chest X-rays and BHM to Breast Histology Microscopy slides. Note that here we provide the total number of classes available for the datasets. This does not mean, however, that all class labels are used. For example, CheXpert is treated as Pleural Effusion many-to-one. More details can be found in Section 4.2.

Dataset	Type	# Images	# Classes
CheXpert	CXR	224,316	14
Shenzhen-CXR	CXR	662	2
Montgomery-CXR	CXR	138	2
ChestX-ray14	CXR	112,120	14
BACH	BHM	400	4
EyePACS	Fundus	35,126	5
iChallenge-AMD	Fundus	400	2
iChallenge-PM	Fundus	400	2

**Table 2:** Summary of terminology used for datasets and models.

	Models
Supervised	Supervised pretrained on ImageNet *(ResNet-50, ResNet-18, DenseNet-121)*
Self-supervised	Self-Supervised pretrained on ImageNet *(SimCLR-v1, MoCo-v2, PIRL, SwAV, BYOL)*
Domain-specific Self-Supervised	Self-Supervised pretrained on chest X-rays *(MIMIC-CheXpert, MoCo-CXR)*
	**Datasets**
In-domain	All chest X-ray datasets *(CheXpert, Shenzhen-CXR Montgomery-CXR, ChestX-ray14)*
Out-of-domain	All non chest X-ray datasets *(BACH, EyePACS, iChallenge-AMD, iChallenge-PM)*

**Table 3:** Few-shot shot transfer performance of the pretrained models on the different medical datasets. All are evaluated as 2-way 20-shot, except ChestX and EyePACS, which are 5-way 20-shot. Results are reported as average accuracy over 600 episodes with 95% CI. Key: **best**, second best.

	CheXpert	Shenzhen	Montgomery	ChestX	BACH	EyePACS	iC-AMD	iC-PM
SimCLR-v1	59.73 ± 0.72	74.46 ± 0.66	63.20 ± 0.80	29.86 ± 0.46	80.61 ± 0.74	32.78 ± 0.42	47.90 ± 0.62	94.92 ± 0.32
MoCo-v2	57.39 ± 0.75	73.76 ± 0.66	63.54 ± 0.74	28.69 ± 0.44	82.53 ± 0.71	34.07 ± 0.43	74.91 ± 0.64	94.21 ± 0.33
SwAV	57.61 ± 0.77	75.22 ± 0.65	67.38 ± 0.71	27.76 ± 0.44	82.78 ± 0.65	**34.47 ± 0.43**	70.94 ± 0.66	94.69 ± 0.31
BYOL	58.44 ± 0.74	76.29 ± 0.65	**70.98 ± 0.67**	30.28 ± 0.46	**83.28 ± 0.66**	33.66 ± 0.41	74.58 ± 0.61	**95.83 ± 0.28**
PIRL	58.51 ± 0.76	**77.48 ± 0.60**	63.58 ± 0.76	28.52 ± 0.44	81.02 ± 0.69	34.19 ± 0.41	75.26 ± 0.60	93.49 ± 0.35
Supervised (r50)	56.14 ± 0.76	70.86 ± 0.72	62.31 ± 0.75	27.71 ± 0.46	80.49 ± 0.68	31.32 ± 0.43	75.70 ± 0.64	94.80 ± 0.33
Supervised (r18)	57.69 ± 0.80	74.16 ± 0.66	62.94 ± 0.69	28.58 ± 0.40	80.78 ± 0.71	32.96 ± 0.41	74.59 ± 0.62	93.68 ± 0.35
Supervised (d121)	57.41 ± 0.78	73.43 ± 0.65	65.31 ± 0.67	27.88 ± 0.44	81.21 ± 0.70	33.49 ± 0.42	**77.12 ± 0.60**	94.86 ± 0.30
MIMIC-CheXpert	**62.45 ± 0.75**	73.22 ± 0.64	69.15 ± 0.66	**34.82 ± 0.48**	71.60 ± 0.98	25.71 ± 0.40	65.05 ± 0.72	83.66 ± 0.54
MoCo-CXR	60.33 ± 0.74	73.89 ± 0.64	65.02 ± 0.70	29.01 ± 0.46	69.07 ± 0.82	27.78 ± 0.41	68.17 ± 0.72	87.59 ± 0.47

**Table 4:** Many-shot shot transfer performance of the pretrained models on the different medical datasets. For EyePACS we do not perform many-shot recognition (linear or finetune) with the domain-specific models (MIMIC-CheXpert, MoCo-CXR), as each run takes over 24 h and we anticipate very poor results, as with few-shot. Key: **best**, second best.

	Linear		Finetune	
	CheXpert	EyePACS	CheXpert	EyePACS
SimCLR	75.01	31.51	75.82	36.48
MoCo	74.94	32.18	**79.96**	45.64
SwAV	74.97	**37.61**	77.84	44.47
BYOL	74.61	34.27	78.97	41.17
PIRL	74.20	31.51	78.30	40.89
Supervised (r50)	73.47	31.46	79.43	**47.08**
Supervised (r18)	71.43	30.52	79.31	42.66
Supervised (d121)	72.50	33.96	79.62	40.18
MIMIC-CheXpert	**77.28**		78.80	
MoCo-CXR	74.76		74.98	

**Figure A1:**
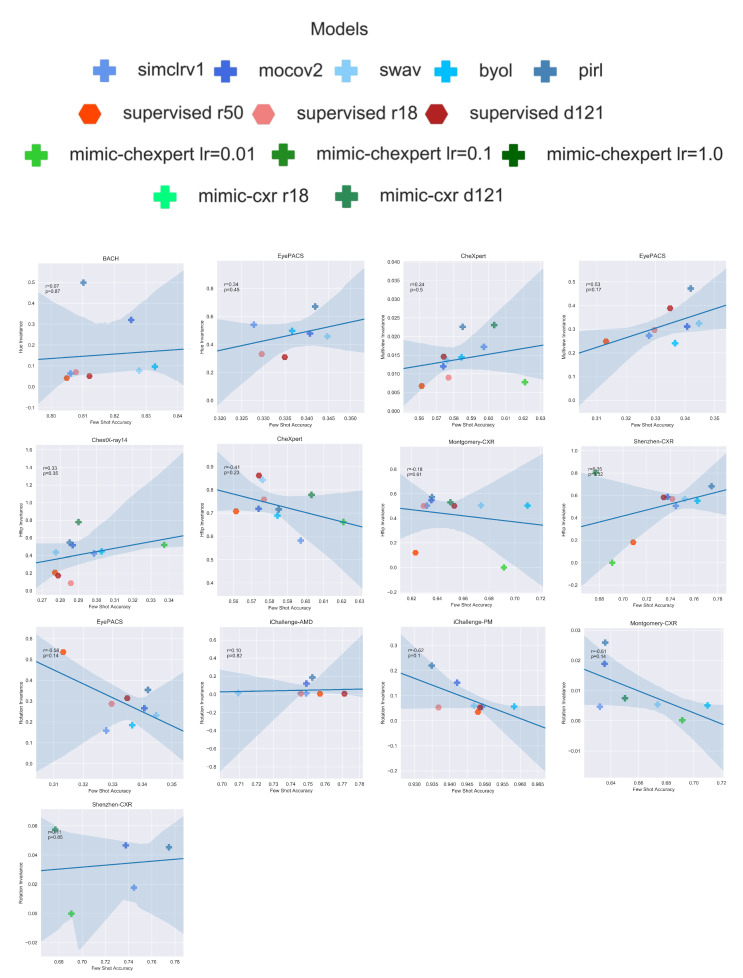
Invariances, as measured by cosine similarity (Equation (3)), plotted against few-shot performance for the different medical datasets. The invariances plotted include rotation, horizontal flip, hue transform and multi-view (the invariance considered in each plot can be found in the y-axis label). Shown overlaid are the Pearson’s *r* correlation coefficients and associated *p* values. We find no significant correlations for any of the plots (high *p* values), except for multi-view invariance on CheXpert, which shows a strong positive correlation.

**Figure A4:**
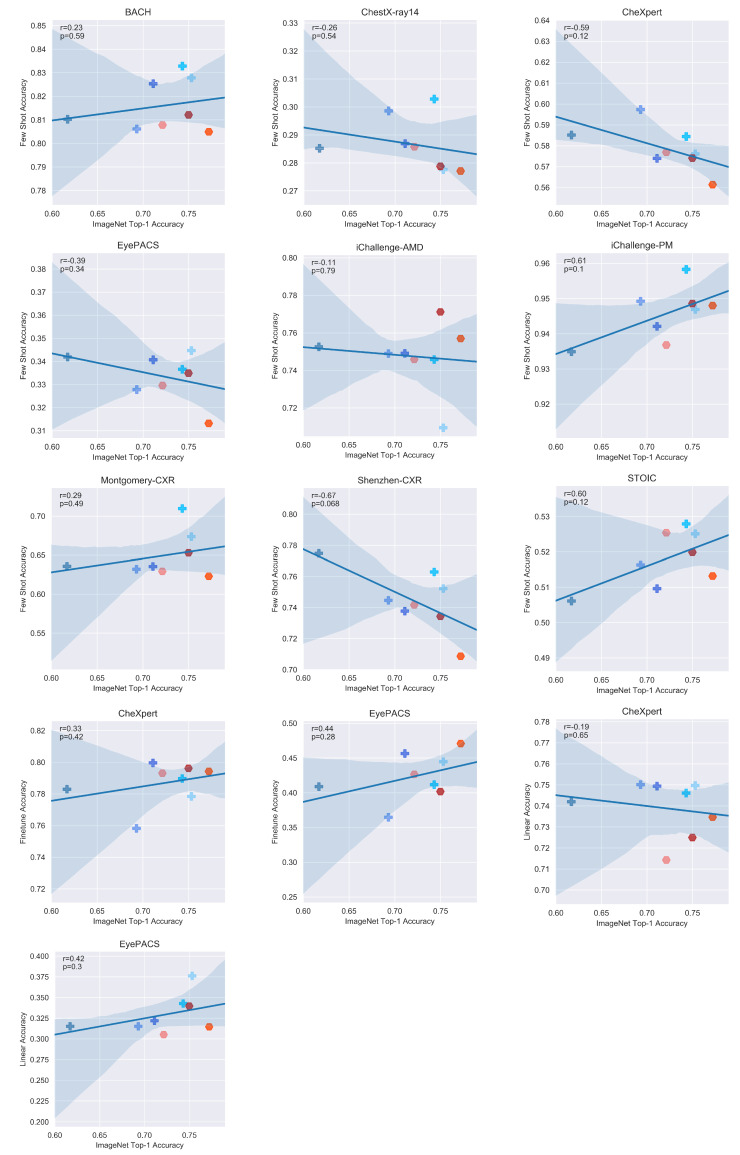
ImageNet top-1 accuracies plotted against transfer performance. Shown overlaid are the Pearson’s *r* correlation coefficients and associated *p* values.

**Figure A5:**
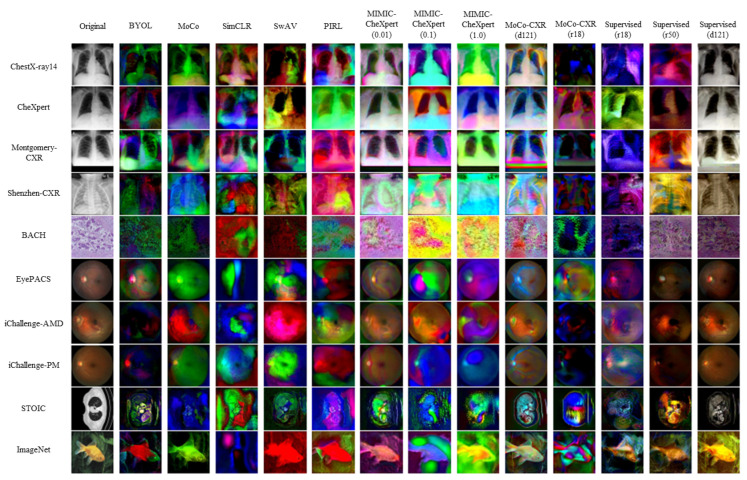
Deep image prior reconstructions for all models on one image from each dataset.

## Data Availability

Publicly available datasets were analyzed in this study. This data can be found here: BACH https://zenodo.org/record/3632035 (accessed on 12 April 2022), ChestX-ray14 https://www.kaggle.com/nih-chest-xrays/data (accessed on 14 April 2022), CheXpert https://stanfordmlgroup.github.io/competitions/chexpert/ (accessed on 6 April 2022), CIFAR10 https://pytorch.org/vision/stable/datasets.html (accessed on 3 April 2022), EyePACS (Diabetic Retinopathy) https://www.kaggle.com/competitions/diabetic-retinopathy-detection/data (accessed on 11 April 2022), iChallenge-AMD https://ai.baidu.com/broad/subordinate?dataset=amd (accessed on 14 April 2022), iChallenge-PM https://ai.baidu.com/broad/subordinate?dataset=pm (accessed on 14 April 2022), Montgomery-CXR https://openi.nlm.nih.gov/faq#faq-tb-coll (accessed on 8 April 2022), Shenzhen-CXR https://openi.nlm.nih.gov/faq#faq-tb-coll (accessed on 8 April 2022), STOIC https://registry.opendata.aws/stoic2021-training/ (accessed on 17 April 2022). No datasets were generated as part of this research.

## Abbreviations

The following abbreviations are used in this manuscript:

SSL	Self-supervised learning
SimCLR	Simple framework for Contrastive Learning of visual representations
	(see Section 2)
PIRL	Pretext-Invariant Representation learning (see Section 2)
MOCO	Momentum Contrast (see Section 2)
BYOL	Bootstrap Your Own Latent (see Section 2)
SwAV	Swapping Assignments Between Views (see Section 2)
CXR	Chest X-rays
BHM	Breast Histology Microscopy slides
MIMIC-CheXpert	Self-supervised model pre-trained on chest X-rays dataset
	(see Section 4.1)
MoCo-CXR	Self-supervised model pre-trained on chest X-rays dataset
	(see Section 4.1)
iChalleng-PM	Pathological Myopia (PM) retinal fundus images dataset (see Section 4.2)
iChallenge-AMD	Age-related Macular degeneration (AMD) retinal images dataset
	(see Section 4.2)
EyePACS	Retinal images dataset for diabetic retinopathy detection (see Section 4.2)
BACH	Breast Cancer Histology images dataset (see Section 4.2)

## Appendix A. Invariances

**Table A1:** Rotational invariance evaluated on the retinal fundus datasets, as well as Hue colour invariance evaluated on the BACH dataset, as measured by cosine similarity. Key: **best**, second best.

	EyePACS	iChallenge-AMD	iChallenge-PM	BACH
SimCLR	0.159	0.011	0.056	0.063
MoCo	0.266	0.120	0.152	0.319
SwAV	0.232	0.015	0.059	0.077
BYOL	0.185	0.012	0.057	0.097
PIRL	**0.354**	**0.187**	**0.220**	**0.498**

**Table A2:** Horizontal flip invariance evaluated on the chest X-ray datasets, as measured by cosine similarity. Key: **best**, second best.

	CheXpert	ChestX	Shenzhen	Montgomery
SimCLR	0.582	0.423	0.506	0.503
MoCo	0.719	0.517	0.587	0.547
SwAV	0.843	0.433	0.569	0.505
BYOL	0.689	0.445	0.551	0.504
PIRL	0.716	0.547	0.682	**0.574**
MIMIC-CheXpert	0.662	0.518	0.001	0.003
MoCo-CXR	**0.997**	**0.778**	**0.803**	0.530

**Table A3:** Multi-view invariance evaluated on the CheXpert and EyePACS datasets, as measured by cosine similarity. Key: **best**, second best.

	CheXpert	EyePACS
SimCLR	0.017	0.273
MoCo	0.012	0.312
SwAV	0.014	0.325
BYOL	0.014	0.241
PIRL	0.023	**0.472**
MIMIC-CheXpert	0.008	
MoCo-CXR	**0.024**	

## Appendix B. Perceptual Distances

**Table A4:** Perceptual distance values, as measured by the LPIPS (Learned Perceptual Image Patch Similarity) metric using the AlexNet architecture [47], for the reconstructed images for each model for each dataset. A low value close to 0 indicates a strong perceptual similarity between the original and reconstructed images.

	Shenzhen	Montomery	EyePACS	ChestX	BACH	iC-AMD	iC-PM	CheXpert	ImageNet
SimCLR	0.95	0.80	0.75	0.80	0.92	0.76	0.86	0.78	0.78
MoCo	0.79	0.74	0.72	0.70	0.74	0.54	0.77	0.70	0.50
SwAV	0.90	0.75	0.78	0.77	0.85	0.64	0.90	0.76	0.74
BYOL	0.96	0.76	0.66	0.76	0.80	0.73	0.72	0.77	0.60
PIRL	0.80	0.76	0.66	0.68	0.66	0.63	0.64	0.79	0.62
Supervised (r50)	0.84	0.89	0.47	0.76	0.37	0.46	0.50	0.80	0.51
Supervised (r18)	0.90	0.76	0.55	0.80	0.64	0.68	0.84	0.82	0.56
Supervised (d121)	0.51	0.45	0.33	0.34	0.49	0.22	0.19	0.23	0.48
MIMIC-CheXpert	0.79	0.70	0.71	0.81	0.79	0.74	0.84	0.72	0.75
MoCo-CXR	0.79	0.64	0.68	0.72	0.72	0.68	0.71	0.75	0.69

**Table A5:** Perceptual distance values, as measured by the LPIPS (Learned Perceptual Image Patch Similarity) metric using the SqueezeNet architecture, for the reconstructed images for each model for each dataset. A low value close to 0 indicates a strong perceptual similarity between the original and reconstructed images.

	Shenzhen	Montomery	EyePACS	ChestX	BACH	iC-AMD	iC-PM	CheXpert	ImageNet
SimCLR	0.92	0.77	0.64	0.79	0.87	0.72	0.73	0.69	0.80
MoCo	0.67	0.67	0.60	0.67	0.71	0.43	0.50	0.61	0.43
SwAV	0.83	0.68	0.65	0.81	0.65	0.56	0.71	0.75	0.76
BYOL	0.84	0.65	0.51	0.74	0.61	0.58	0.58	0.71	0.50
PIRL	0.85	0.76	0.52	0.66	0.56	0.50	0.49	0.68	0.59
Supervised (r50)	0.89	0.89	0.34	0.81	0.26	0.32	0.38	0.78	0.38
Supervised (r18)	0.79	0.73	0.46	0.76	0.47	0.57	0.68	0.79	0.46
Supervised (d121)	0.53	0.40	0.22	0.32	0.27	0.14	0.14	0.40	0.39
MIMIC-CheXpert	0.73	0.64	0.62	0.75	0.80	0.63	0.72	0.76	0.73
MoCo-CXR	0.88	0.60	0.63	0.72	0.65	0.62	0.61	0.78	0.64

**Table A6:** Perceptual distance values, as measured by the LPIPS (Learned Perceptual Image Patch Similarity) metric using the VGG architecture, for the reconstructed images for each model for each dataset. A low value close to 0 indicates a strong perceptual similarity between the original and reconstructed images.

	Shenzhen	Montomery	EyePACS	ChestX	BACH	iC-AMD	iC-PM	CheXpert	ImageNet
SimCLR	0.84	0.79	0.74	0.81	0.92	0.88	0.90	0.79	0.89
MoCo	0.73	0.77	0.91	0.81	0.87	0.64	0.83	0.70	0.64
SwAV	0.84	0.73	0.83	0.80	0.83	0.75	0.96	0.86	0.93
BYOL	0.79	0.78	0.73	0.78	0.82	0.74	0.82	0.76	0.74
PIRL	0.77	0.76	0.72	0.72	0.79	0.77	0.76	0.89	0.78
Supervised (r50)	0.88	0.89	0.56	0.77	0.59	0.51	0.57	0.81	0.61
Supervised (r18)	0.75	0.76	0.61	0.77	0.65	0.75	0.85	0.91	0.67
Supervised (d121)	0.57	0.56	0.46	0.45	0.57	0.35	0.32	0.19	0.59
MIMIC-CheXpert	0.82	0.85	0.84	0.91	1.00	0.79	0.89	0.86	0.87
MoCo-CXR	0.79	0.69	0.87	0.75	0.81	0.73	0.79	0.82	0.83

## Appendix C. Attentive Diffusions

**Table A7:** Attentive diffusion values for the saliency maps for each model for each dataset. A low value close to 0 implies a narrow focus. The attentive diffusion corresponds to the proportion of the saliency map with value above the mean [20].

	Shenzhen	Montomery	EyePACS	ChestX	BACH	iC-AMD	iC-PM	CheXpert	ImageNet
SimCLR	0.47	0.51	0.45	0.39	0.55	0.46	0.47	0.39	0.26
MoCo	0.40	0.43	0.44	0.46	0.43	0.40	0.45	0.49	0.41
SwAV	0.48	0.54	0.48	0.53	0.48	0.42	0.48	0.52	0.49
BYOL	0.39	0.41	0.47	0.41	0.41	0.36	0.42	0.50	0.39
PIRL	0.45	0.49	0.43	0.49	0.48	0.45	0.50	0.49	0.47
Supervised (r50)	0.42	0.44	0.40	0.34	0.43	0.43	0.46	0.47	0.43
Supervised (r18)	0.43	0.43	0.36	0.43	0.43	0.39	0.47	0.51	0.35
Supervised (d121)	0.45	0.49	0.47	0.43	0.43	0.41	0.40	0.52	0.40
MIMIC-CheXpert	0.44	0.34	0.48	0.33	0.44	0.45	0.49	0.34	0.45
MoCo-CXR	0.47	0.31	0.47	0.36	0.47	0.50	0.55	0.33	0.42
